# Brian2Loihi: An emulator for the neuromorphic chip Loihi using the spiking neural network simulator Brian

**DOI:** 10.3389/fninf.2022.1015624

**Published:** 2022-11-09

**Authors:** Carlo Michaelis, Andrew B. Lehr, Winfried Oed, Christian Tetzlaff

**Affiliations:** ^1^Department of Computational Neuroscience, University of Göttingen, Göttingen, Germany; ^2^Bernstein Center for Computational Neuroscience, University of Göttingen, Göttingen, Germany

**Keywords:** neuromorphic computing, Loihi, Brian2, emulator, spiking neural network, open source

## Abstract

Developing intelligent neuromorphic solutions remains a challenging endeavor. It requires a solid conceptual understanding of the hardware's fundamental building blocks. Beyond this, accessible and user-friendly prototyping is crucial to speed up the design pipeline. We developed an open source Loihi emulator based on the neural network simulator Brian that can easily be incorporated into existing simulation workflows. We demonstrate errorless Loihi emulation in software for a single neuron and for a recurrently connected spiking neural network. On-chip learning is also reviewed and implemented, with reasonable discrepancy due to stochastic rounding. This work provides a coherent presentation of Loihi's computational unit and introduces a new, easy-to-use Loihi prototyping package with the aim to help streamline conceptualization and deployment of new algorithms.

## 1. Introduction

Neuromorphic computing offers exciting new computational structures. Decentralized units inspired by neurons are implemented in hardware (reviewed by Schuman et al., 2017; Rajendran et al., 2019; Young et al., 2019). These can be connected up to one another, stimulated with inputs, and the resulting activity patterns can be read out from the chip as output. A variety of algorithms and applications have been developed in recent years, including robotic control (DeWolf et al., 2016, 2020; Michaelis et al., 2020; Stagsted et al., 2020), spiking variants of deep learning algorithms, attractor networks, nearest-neighbor or graph search algorithms (reviewed by Davies et al., 2021). Moreover, neuromorphic hardware may provide a suitable substrate for performing large scale simulations of the brain (Furber, 2016; Thakur et al., 2018). Neuromorphic chips specialized for particular computational tasks can either be provided as a neuromorphic computing cluster or be integrated into existing systems, akin to graphics processing units (GPU) in modern computers (Furber et al., 2014; Davies et al., 2021). With the right ideas, networks of spiking units implemented in neuromorphic hardware can provide the basis for powerful and efficient computation. Nevertheless, the development of new algorithms for spiking neural networks, applicable to neuromorphic hardware, is a challenge (Grüning and Bohte, 2014; Pfeiffer and Pfeil, 2018; Bouvier et al., 2019).

At this point, without much background knowledge of neuromorphic hardware, one can get started programming using the various software development kits available (e.g., Brüderle et al., 2011; Sawada et al., 2016; Lin et al., 2018; Rhodes et al., 2018; Michaelis, 2020; Müller et al., 2020a,b; Spilger et al., 2020; Rueckauer et al., 2021). Emulators for neuromorphic hardware (Furber et al., 2014; Petrovici et al., 2014; Luo et al., 2018; Valancius et al., 2020) running on a standard computer or field programmable gate arrays (FPGA), make it possible to develop neuromorphic network architectures without even needing access to a neuromorphic chip (see e.g., NengoLoihi^1^ and Dynap-SE^2^). This can speed up prototyping as the initialization of networks, i.e., distributing neurons and synapses, as well as the readout of the system's state variables on neuromorphic chips takes some time. At the same time emulators transparently contain the main functionalities of the hardware in code and therefore provide insights into how it works. With this understanding, algorithms can be intelligently designed and complex network structures implemented.

In the following, we introduce an emulator for the digital neuromorphic chip Loihi (Davies et al., 2018) based on the widely used spiking neural network simulator Brian (Stimberg et al., 2019). We first dissect an individual computational unit from Loihi. The basic building block is a spiking unit inspired by a current based leaky integrate and fire (LIF) neuron model (see Gerstner et al., 2014). Connections between these units can be plastic, enabling the implementation of diverse on-chip learning rules. Analyzing the computational unit allows us to create an exact emulation of the Loihi hardware on the computer. We extend this to a spiking neural network model and demonstrate that both Loihi and Brian implementations match perfectly. This exact match means one can do prototyping directly on the computer using Brian only, which adds another emulator in addition to the existing simulation backend in the Nengo Loihi library. This increases both availability and simplicity of algorithm design for Loihi, especially for those who are already used to working with Brian. In particular for the computational neuroscience community, this facilitates the translation of neuroscientific models to neuromorphic hardware. Finally, we review and implement synaptic plasticity and show that while individual weights show small deviations due to stochastic rounding, the statistics of a learning rule are preserved. Our aim is to facilitate the development of neuromorphic algorithms by delivering an open source emulator package that can easily be incorporated into existing workflows. In the process we provide a solid understanding of what the hardware computes, laying the appropriate foundation to design precise algorithms from the ground up.

## 2. Loihi's computational unit and its implementation

Developing a Loihi emulator requires precise understanding of how Loihi works. And to understand how something works, it is useful to “take it apart and put it back together again”. While we will not physically take the Loihi chip apart, we can inspect the components of its computational units with “pen and paper”. Then, by implementing each component on a computer we will test that, when put back together, the parts act like we expect them to. In the following we highlight how spiking units on Loihi approximate a variant of the well-known LIF model using first order Euler numerical integration with integer precision. This understanding enables us to emulate Loihi's spiking units on the computer in a way that is straightforward to use and easy to understand. For a better intuition of how the various parameters on Loihi interact, we refer readers to our neuron design tool^3^ for Loihi. Readers familiar with Davies et al. (2018) and numerical implementations of LIF neurons may prefer to skip to Section 2.3.

### 2.1. Loihi's neuron model: A recap

The basic computational unit on Loihi is inspired by a spiking neuron (Davies et al., 2018). Loihi uses a variant of the leaky integrate and fire neuron model (Gerstner et al., 2014) (see Appendix 9.1). Each unit *i* of Loihi implements the dynamics of the voltage *v*_*i*_


(1) $$\begin{array}{l} \frac{dv_{i}}{dt}=-\frac{1}{\tau_{v}}v_{i}\left(t\right)+I_{i}\left(t\right)-v_{i}^{th}\sigma_{i}\left(t\right), \end{array}$$

where the first term controls the voltage decay, the second term is the input to the unit, and the third term resets the voltage to zero after a spike by subtracting the threshold. A spike is generated if $v_{i}>v_{i}^{th}$ and transmitted to other units to which unit *i* is connected. In particular, *v* models the voltage across the membrane of a neuron, τ_*v*_ is the time constant for the voltage decay, *I* is an input variable, *v*^*th*^ is the threshold voltage to spike, and σ(*t*) is the so-called spike train which is meant to indicate whether the unit spiked at time *t*. For each unit *i*, σ_*i*_(*t*) can be written as a sum of Dirac delta distributions


(2) $$\begin{array}{l} \sigma_{i}\left(t\right)=\underset{k}{\sum }\delta \left(t-t_{i,k}\right), \end{array}$$

where *t*_*i, k*_ denotes the time of the *k*-th spike of unit *i*. Note that σ_*i*_ is not a function, but instead defines a *distribution* (i.e., *generalized function*), and is only meaningful under an integral sign. It is to be understood as the linear functional $\langle \sigma_{i},f\rangle :=\int \sigma_{i}\left(t\right)f\left(t\right)dt=\underset{k}{\sum }f\left(t_{i,k}\right)$ for arbitrary, everywhere-defined function *f* (see Corollary 1 in Appendix 9.1.2).

Input to a unit can come from user defined external stimulation or from other units implemented on chip. Davies et al. (2018) describe the behavior of the input *I*(*t*) with


(3) $$\begin{array}{l} I_{i}\left(t\right)=\underset{j}{\sum }J_{ij}\left(\alpha_{I}*\sigma_{j}\right)\left(t\right)+I_{i}^{\text{bias}}, \end{array}$$

where *J*_*ij*_ is the weight from unit *j* to *i*, $I_{i}^{\text{bias}}$ is a constant bias input, and the spike train σ_*j*_ of unit *j* is convolved with the synaptic filter impulse response α_*I*_, given by


(4) $$\begin{array}{l} \alpha_{I}\left(t\right)=\exp\left(-\frac{t}{\tau_{I}}\right)H\left(t\right), \end{array}$$

where τ_*I*_ is the time constant of the synaptic response and *H*(*t*) the unit step function. Note that α_*I*_(*t*) is defined differently here than in Davies et al. (2018) (see Appendix 9.1.3 for details). The convolution from Equation (3) is a notational convenience for defining the synaptic input induced by an incoming spike train, simply summing over the time-shifted synaptic response functions, namely $\left(\sigma_{i}*f\right)\left(t\right)=\langle \sigma_{i},\tau_{t}\tilde{f}\rangle =\underset{k}{\sum }f\left(t-t_{i,k}\right)$, where τ_*t*_*f*(*x*) = *f*(*x*−*t*) and $\tilde{f}\left(x\right)=f\left(-x\right)$ (see Appendix 9.1.2).

### 2.2. Implementing Loihi's spiking unit in software

From the theoretical model on which Loihi is based, we can derive the set of operations each unit implements with a few simple steps. Using a first order approximation for the differential equations gives the update equations for the voltage and synaptic input described in the Loihi documentation.^4^ Combined with a few other details regarding Loihi's integer precision and the order of operations, we will have all we need to implement a Loihi spiking unit in software.

#### 2.2.1. Synaptic input

From Equation (3), we see that the synaptic input can be written as a sum of exponentially decaying functions with amplitude *J*_*ij*_ beginning at the time of each spike *t*_*j, k*_ (see Appendix 9.1.2). In particular we have


(5) $$\begin{array}{l} I_{i}\left(t\right)=\underset{j}{\sum }J_{ij}\underset{k}{\sum }\exp\left(\frac{t_{j,k}-t}{\tau_{I}}\right)H\left(t-t_{j,k}\right)+I_{i}^{\text{bias}}. \end{array}$$

To understand the behavior of the synaptic input it is helpful to consider the effect of one spike arriving at a single synapse. Simplifying Equation (5) to just one neuron that receives just one input spike at time *t*_1_ = 0, for *t* ≥ 0 we get


(6) $$\begin{array}{l} I\left(t\right)=J\cdot \exp\left(-\frac{t}{\tau_{I}}\right) \end{array}$$

and for *t* < 0, *I*(*t*) = 0. Each spike induces a step increase in the current which decays exponentially with time constant τ_*I*_. Taking the derivative of both sides with respect to *t* gives


(7) $$\begin{array}{ll} \frac{dI}{dt} & =-\frac{1}{\tau_{I}}\cdot I\left(t\right), \end{array}$$

(8) $$\begin{array}{ll} I\left(0\right) & =J. \end{array}$$

Applying the forward Euler method to the differential equation for Δ*t* = 1 and *t* ≥ 0, *t* ∈ ℕ we get


(9) $$\begin{array}{l} I\left[t\right]=I\left[t-1\right]-\frac{1}{\tau_{I}}\cdot I\left[t-1\right]+J\cdot s\left[t\right], \end{array}$$

where *s*[*t*] is zero unless there is an incoming spike on the synapse, in which case it is one. Here, *s*[0] = 1 and *s*[*t*] = 0 for *t* > 0. With this we have simply incorporated the initial condition into the update equation. Note that we have switched from a continuous [e.g., *I*(*t*)] to discrete (e.g., *I*[*t*]) time formulation, where Δ*t* = 1 and *t* is unitless.

Loihi has a decay value δ^*I*^, which is inversely proportional to τ_*I*_, namely $\delta^{I}=2^{12}/\tau_{I}$. Swapping τ_*I*_ by δ^*I*^ reveals


(10) $$\begin{array}{l} I\left[t\right]=I\left[t-1\right]\cdot \left(2^{12}-\delta^{I}\right)\cdot 2^{-12}+J\cdot s\left[t\right]. \end{array}$$

The weight *J* is defined *via* the mantissa ${\tilde{w}}_{ij}$ and exponent Θ (see Section 3.1) such that the equation describing the synaptic input becomes (with indices)


(11) $$\begin{array}{l} I_{i}\left[t\right]=I_{i}\left[t-1\right]\cdot \left(2^{12}-\delta^{I}\right)\cdot 2^{-12}+2^{6+\Theta }\cdot \underset{j}{\sum }\left({\tilde{w}}_{ij}\cdot s_{j}\left[t\right]\right), \end{array}$$

where *s*_*j*_[*t*] ∈ {0, 1} is the spike state of the *j*^*th*^ input neuron. Please note that Equation (2.2.1) is identical to the Loihi documentation.

**Figure 1:**
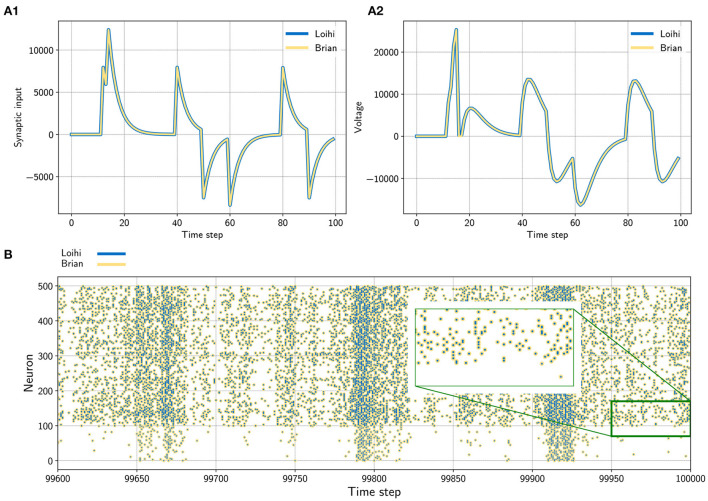
**(A)** Input trace of a single synapse (left) and voltage trace (right) of a neuron. The neuron receives randomly timed excitatory and inhibitory input spikes. The emulator (yellow) matches Loihi (blue) in both cases perfectly. Note that Loihi uses the voltage register to count refractory time, which results in a functionally irrelevant difference after a spike, e.g time step 17 in A2 (see Appendix 9.2.2). **(B)** Network simulation with 400 excitatory (indices 100 − 500) and 100 inhibitory (indices 0 − 100) neurons. The network is driven by noise from an input population of 40 Poisson spike generators with a connection probability of 0.05. All spikes match exactly between the emulator and Loihi for all time steps. The figure shows the last 400 time steps from a simulation with 100, 000 time steps.

From this we can conclude that the implementation of synaptic input on Loihi is equivalent to evolving the LIF synaptic input differential equation with the forward Euler numerical integration method (see Figure 1A1).

#### 2.2.2. Voltage

It is straightforward to perform the same analysis as above for the voltage equation. We consider the subthreshold voltage dynamics for a single neuron and can therefore ignore the reset term $v_{i}^{th}\sigma_{i}\left(t\right)$ from Equation (1), leaving us with


(12) $$\begin{array}{l} \frac{dv}{dt}=-\frac{1}{\tau_{v}}v\left(t\right)+I\left(t\right). \end{array}$$

Applying forward Euler gives


(13) $$\begin{array}{l} v\left[t\right]=v\left[t-1\right]-\frac{v\left[t-1\right]}{\tau_{v}}+I\left[t\right]. \end{array}$$

Again, to compare with the Loihi documentation we need to swap the time constant τ_*v*_ by a voltage decay parameter, δ^*v*^, which is inversely proportional to the time constant, the same as above for synaptic input. Plugging in $\tau_{v}=2^{12}/\delta^{v}$ leads to


(14) $$\begin{array}{l} v\left[t\right]=v\left[t-1\right]\cdot \left(2^{12}-\delta^{u}\right)\cdot 2^{-12}+I\left[t\right]. \end{array}$$

By introducing a bias term, the voltage update becomes


(15) $$\begin{array}{l} v_{i}\left[t\right]=v_{i}\left[t-1\right]\cdot \left(2^{12}-\delta^{u}\right)\cdot 2^{-12}+I_{i}\left[t\right]+I_{i}^{\text{bias}}. \end{array}$$

Equation (15) agrees with the Loihi documentation. Like the synaptic input, the voltage implementation on Loihi is equivalent to updating the LIF voltage differential equation using forward Euler numerical integration (see Figure 1A2).

#### 2.2.3. Integer precision

Loihi uses integer precision. So the mathematical operations in the update equations above are to be understood in terms of integer arithmetic. In particular, for the synaptic input and voltage equations the emulator uses *round away from zero*, which can be defined as


(16) $$\begin{array}{l} x_{\text{round}}: =\mathrm{sign}\left(x\right)\cdot \left\lceil \left|x\right|\right\rceil . \end{array}$$

where ⌈·⌉ is the ceiling function and sign(·) the sign function.

### 2.3. Summary

We now have all of the pieces required to understand and emulate a spiking unit from Loihi. Evolving the differential equations for the current-based LIF model with the forward Euler method and using the appropriate rounding (see Section 2.2.3) and update schedule (see Section 4.1 and Appendix 9.2.1) is enough to exactly reproduce Loihi's behavior. This procedure is summarized in Algorithm 1 and an exact match between Loihi and an implementation for a single unit in Brian is shown in Figure 1A. Please note that during the refractory period Loihi uses the voltage trace to count elapsed time (see Figure 1A2, Appendix 9.2.2), while in the emulator the voltage is simply clamped to zero.

**Algorithm 1:**
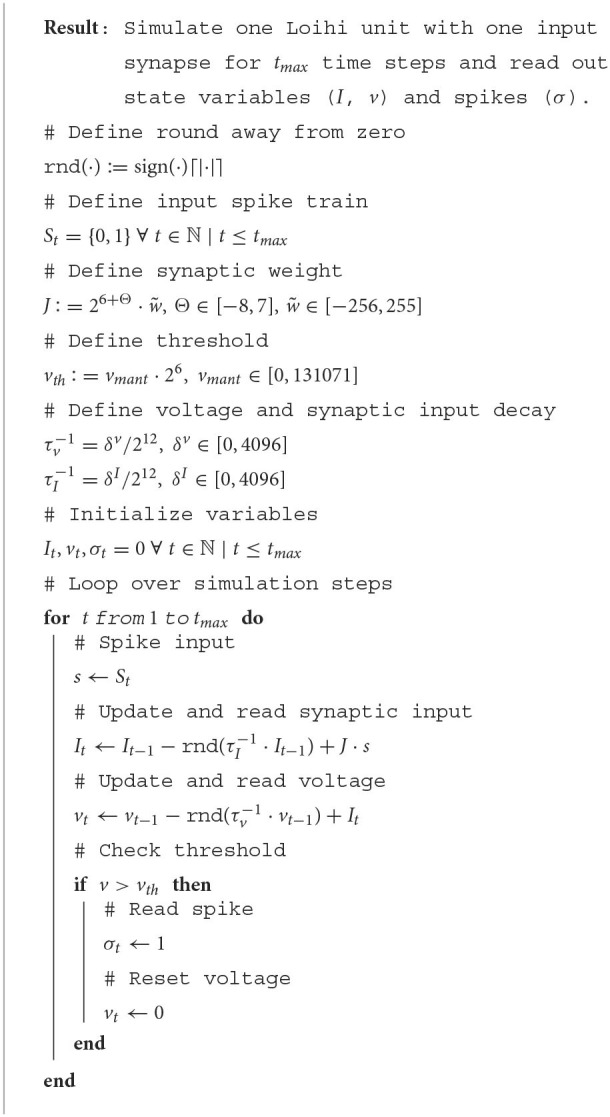
Loihi single neuron emulator.

## 3. Network and plasticity

We now have a working implementation of Loihi's spiking unit. In the next step, we need to connect these units up into networks. And if the network should be able to learn online, connections between units should be plastic. In this section, we review how weights are defined on Loihi and how learning rules are applied. This includes the calculation of pre- and post-synaptic traces. Based on this, we outline how these features are implemented in the emulator.

### 3.1. Synaptic weights

The synaptic weight consists of two parts, a weight mantissa $\tilde{w}$ and a weight exponent Θ and is of the form $\tilde{w}\cdot 2^{6+\Theta }$. However, in practice the calculation of the synaptic weight depends on bit shifts and its precision depends on a few parameters (see below). The weight exponent is a value between −8 and 7 that scales the weight mantissa exponentially. Depending on the sign mode of the weight (excitatory, inhibitory, or mixed), the mantissa is an integer in the range $\tilde{w}\in \left[0,255\right]$, $\tilde{w}\in \left[-255,0\right]$, or $\tilde{w}\in \left[-256,254\right]$, respectively. The possible values of the mantissa depend on the number of bits available for storing the weight and whether the sign mode is *mixed* or not. In particular, precision is defined as $2^{n_{s}}$, with


(17) $$\begin{array}{l} n_{s}=8-\left(n_{wb}-\sigma_{\text{mixed}}\right). \end{array}$$

This can intuitively be understood with a few examples. If the weight bits for the weight mantissa are set to the default value of *n*_*wb*_ = 8 bits, it can store 256 values between 0 and 255, i.e., the precision is then 2^8−(8 − 0)^ = 2^0^ = 1. If *n*_*wb*_ = 6 bits is chosen, we instead have a precision of 2^8−(6 − 0)^ = 2^2^ = 4 meaning there are 64 possible values for the weight mantissa, $\tilde{w}\in \left\{0,4,8,16,...,252\right\}$. If the sign mode is *mixed*, i.e., σ_*mixed*_ = 1, one bit is used to store the sign, which reduces the precision. Mixed mode enables both positive and negative weights, with weight mantissa between −256 and 254. Assuming *n*_*wb*_ = 8 in mixed mode, precision is 2^8−(8 − 1)^ = 2^1^ = 2 and $\tilde{w}\in \left\{-256,-254,...,-4,-2,0,2,4,...,254\right\}$.

#### 3.1.1. Weight initialization

While the user can define an arbitrary weight mantissa within the allowed range, during initialization the value is rounded, given the precision, to the next possible value toward zero. This is achieved *via* bit shifting, that is the weight mantissa is shifted by


(18) $$\begin{array}{l} {\tilde{w}}^{\text{shifted}}=\left(\tilde{w}\gg n_{s}\right)\ll n_{s}, \end{array}$$

where ≫ and ≪ are a right and left shift respectively. Afterwards the weight exponent is used to scale the weight according to


(19) $$\begin{array}{l} J^{\text{scaled}}={\tilde{w}}^{\text{shifted}}\cdot 2^{6+\Theta }. \end{array}$$

This value cannot be greater than 21 bits and is clipped if it exceeds this limit. Note that this only happens in one case for $\tilde{w}=-256$ and Θ = 7. Finally the scaled value *J*^scaled^ is shifted again according to


(20) $$\begin{array}{l} J=\left(J^{\text{scaled}}\gg 6\right)\ll 6, \end{array}$$

where *J* is the final weight.

We provide a table with all 4096 possible weights depending on the mantissa and the exponent in a Jupyter notebook^5^. These values are provided for all three sign modes.

#### 3.1.2. Plastic synapses

In the case of a *static* synapse, the initialized weight remains the same as long as the chip/emulator is running. Thus *static* synapses are fully described by the details above. For *plastic* synapses, the weight can change over time. This requires a method to ensure that changes to the weight adhere to its precision.

For *plastic* synapses, *stochastic rounding* is applied to the mantissa during each weight update. Whether the weight mantissa is rounded up or down depends on its proximity to the nearest possible values above and below, i.e.,


(21) $$\begin{array}{l} {\text{RS}}_{2^{n_{s}}}(x)=\\ \{\begin{array}{l} \text{sign}(x)\;\cdot \;{\left\lfloor \left|x\right|\right\rfloor }_{2^{n_{s}}}\text{ with probability }(2^{n_{s}}-(\left|x\right|-{\left\lfloor \left|x\right|\right\rfloor }_{2^{n_{s}}}\text{​}))/2^{n_{s}}\\ \text{sign}(x)\;\cdot \;\left({\left\lfloor \left|x\right|\right\rfloor }_{2^{n_{s}}}+2^{ns}\text{​}\right)\text{ with probability }(\left|x\right|-{\left\lfloor \left|x\right|\right\rfloor }_{2^{n_{s}}}\text{​}))/2^{n_{s}} \end{array} \end{array}$$

where ${\left\lfloor \cdot \right\rfloor }_{2^{n_{s}}}$ denotes rounding down to the nearest multiple of $2^{n_{s}}$. After the mantissa is rounded, it is scaled by the weight exponent and the right/left bit shifting is applied to the result to compute the actual weight *J*. How this is realized in the emulator is shown in Code Listing 3.

To test that our implementation of the weight update for *plastic* synapses matches Loihi for each possible number of weight bits, we compared the progression of the weights over time for a simple learning rule. The analysis is described in detail in Appendix 9.4.

### 3.2. Pre- and post-synaptic traces

Pre- and post-synaptic traces are used for defining learning rules. Loihi provides two pre-synaptic traces *x*_1_, *x*_2_ and three post-synaptic traces *y*_1_, *y*_2_, *y*_3_. Pre-synaptic traces are increased by a constant value ${\hat{x}}_{i}$, for *i* ∈ {1, 2}, if the pre-synaptic neuron spikes. The post-synaptic traces are increased by ŷ_*j*_ for *j* ∈ {1, 2, 3}, accordingly. So-called *dependency factors* are available, indicating events like *x*_0_ = 1 if the pre-synaptic neuron spikes or *y*_0_ = 1 if the post-synaptic neuron spikes. These factors can be combined with the trace variables by addition, subtraction, or multiplication.

A simple spike-time dependent plasticity (STDP) rule with an asymmetric learning window would, for example, look like *dw* = *x*_1_·*y*_0_−*y*_1_·*x*_0_. This rule leads to a positive change in the weight (*dw* > 0) if the pre-synaptic neuron fires shortly before the post-synaptic neuron (i.e., positive trace *x*_1_ > 0 when *y*_0_ = 1) and to a negative change (*dw* < 0) if the post-synaptic neuron fires shortly before the pre-synaptic neuron (i.e., positive trace *y*_1_ > 0 when *x*_0_ = 1). Thus, the time window in which changes may occur depends on the shape of the traces (i.e., impulse strength ${\hat{x}}_{i}$, ŷ_*i*_; and decay τ_*x*_*i*__, τ_*y*_*j*__, see below).

For a sequence of spikes *s*[*t*] ∈ {0, 1}, a trace is defined as


(22) $$\begin{array}{l} x_{i}\left[t\right]=\alpha \cdot x_{i}\left[t-1\right]+{\hat{x}}_{i}\cdot s\left[t\right], \end{array}$$

where α is a decay factor (see Davies et al., 2018). This equation holds for presynaptic (*x*_*i*_) and postsynaptic (*y*_*i*_) traces. However, in practice, on Loihi one does not set α directly but instead decay time constants τ_*x*_*i*__ and τ_*y*_*j*__.

In the implementation of the emulator we again assume a first order approximation for synaptic traces, akin to synaptic input and voltage. Under this assumption for the exponential decay, in Equation (22) we replace α by


(23) $$\begin{array}{l} \alpha \left(\tau_{x_{i}}\right)=1-\frac{1}{\tau_{x_{i}}}. \end{array}$$

Using this approximation gives reasonable results across a number of different τ_*x*_*i*__ and τ_*y*_*i*__ values (see Figure A2). While this essentially suffices, it could be improved by introducing an additional parameter, e.g., β, and optimizing α(τ_*x*_*i*__, β).

Note that we have integer precision again. But different from the *round away from zero* applied in the neuron model, here *stochastic rounding* is used. Since traces are positive values between 0 and 127 with precision 1, the definition above in Equation (21) simplifies to the following


(24) $$\begin{array}{l} {\text{RS}}_{1,\geq 0}\left(x\right)=\{\begin{array}{ll} \left\lfloor x\right\rfloor \; & \text{with probability }1-\left(x-\left\lfloor x\right\rfloor \right)\\ \left\lfloor x\right\rfloor +1\; & \text{with probability }x-\left\lfloor x\right\rfloor \end{array} \end{array}$$

Since this rounding procedure is probabilistic and the details of the random number generator are unknown, rounding introduces discrepancies when emulating Loihi on the computer. Further improvements are possible if more details of the chip's rounding mechanism were to be considered.

### 3.3. Summary

**Figure 2:**
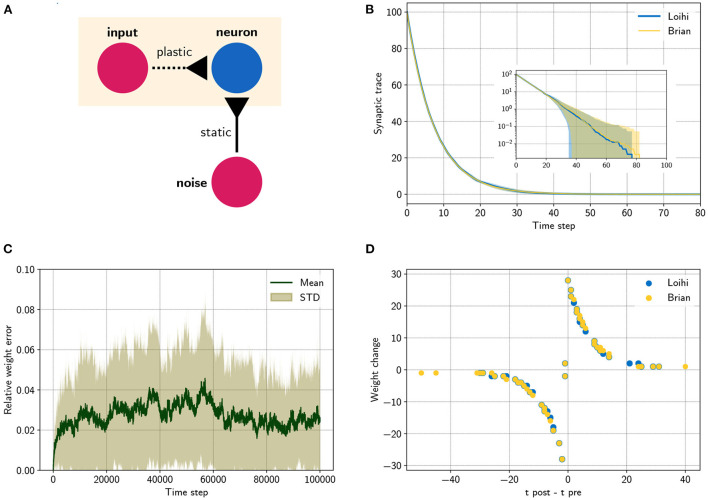
Comparing a STDP learning rule performed with the emulator and with Loihi. **(A)** Sketch showing the setup. **(B)** Synaptic trace for many trials showing the arithmetic mean and standard deviation. The inset shows the same data in a logarithmic scale. Note that every data point smaller than 10^0^ shows the probability of rounding values between 0 and 1 up or down. **(C)** Relative difference $|{\tilde{w}}_{L}-{\tilde{w}}_{B}|/{\tilde{w}}_{\text{max}}$ for the plastic weight between the emulator, ${\tilde{w}}_{B}$, and the Loihi implementation, ${\tilde{w}}_{L}$, for 50 simulations, ${\tilde{w}}_{\text{max}}=255$. **(D)** STDP weight change in respect to pre- and post-synaptic spike times, data shown for time steps 0 − 2, 000 for visualization purposes.

At this point we are able to connect neurons with synapses and build networks of neurons (see Figure 1B). It was shown how the weights are handled, depending on the user defined number of weight bits or the sign mode. In addition, using the dynamics of the pre- and post synaptic traces, we can now define learning rules. Note that different from the neuron model, the synaptic traces cannot be reproduced exactly since the details of the random number generator, used for stochastic rounding, are unknown. However, Figure 2 shows that the synaptic traces emulated in Brian are very close to the original ones in Loihi and that the behavior of a standard asymmetric STDP rule can be reproduced with the emulator.

## 4. Loihi emulator based on Brian

Here we provide an overview over the emulator package and show some examples and results. This enables straightforward emulation of the basic features from Loihi as a sandbox for experimenters. Note that we have explicitly not included routing and mapping restrictions, like limitations for the number of neurons or the amount of synapses, as these depend on constraints such as the number of used Loihi chips.

### 4.1. The package

The emulator package is available on *PyPI*^6^ and can be installed using the pip package manager. The emulator does not provide all functionality of the Loihi chip and software, but the main important aspects. An overview over all provided features is given in Table A1 (Appendix). It contains six classes that extend the corresponding Brian classes. The classes are briefly introduced in the following. Further details can be taken from the code.^7^

#### 4.1.1. Network

The LoihiNetwork class extends the Brian Network class. It provides the same attributes as the original Brian class. The main difference is that it initializes the default clock, the integration methods and updates the schedule when a Network instance is created. Note that it is necessary to make explicitly use of the LoihiNetwork. It is not possible to use Brian's *magic network*.

Voltage and synaptic input are evolved with the forward Euler integration method, which was introduced in Section 2.2. Additionally a state updater was defined for the pre- and post-synaptic traces.

**Figure:**
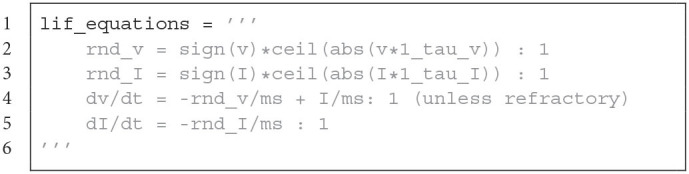
Neuron model equations of the voltage and the synaptic input for Brian. It contains a *round away from zero* rounding.

The default network update schedule for the computational order of the variables from Brian do not match the order of the computation on Loihi. The Brian update schedule is therefore altered when initializing the LoihiNetwork, more details are given in Appendix 9.2.1.

#### 4.1.2. Neuron group

The LoihiNeuronGroup extends Brian's NeuronGroup class. Parameters of the LoihiNeuronGroup class are mostly different from the Brian class and are related to Loihi. When an instance is created, the given parameters are first checked to match requirements from Loihi. Finally, the differential equations to describe the neural system are shown in Code Listing 1. Since Brian does not provide a *round away from zero* functionality, we need to define it manually as an equation.

#### 4.1.3. Synapses

The LoihiSynapses class extends the Synapses class from Brian. Again, most of the Brian parameters are not supported and instead Loihi parameters are available. When instantiating a LoihiSynapses object, the needed pre- and post-synaptic traces are included as equations (shown in Code Listing 2) as theoretically introduced in Section 3.2. Moreover, it is verified that the defined learning rule matches the available variables and operations supported by Loihi. The equations for the weight update is shown in Code Listing 3.

Since we have no access to the underlying mechanism and we cannot reproduce the pseudo-stochastic mechanisms exactly, we have to find a stochastic rounding that matches Loihi in distribution. Note that on Loihi the same network configuration leads to reproducible results (i.e., same rounding). Thus to compare the behavior of Loihi and the emulator, we simulate over a number of network settings and compare the distribution of the traces. Figure 2B shows the match between the distributions. Note that with this, our implementation is always slightly different from the Loihi simulation, due to slight differences in rounding. In Figure 2C, we show that these variations are constant and not diverging. In addition, Figure 2D shows that the principle behavior of a learning rule is preserved.

**Figure:**
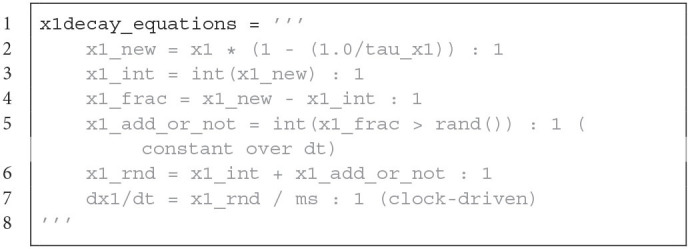
Synaptic decay equation for Brian. Only the decay for *x*1 is shown, the decay for *x*2, *y*1, *y*2, *y*3 is applied analogously. It contains an approximation of the exponential decay and stochastic rounding.

**Figure:**
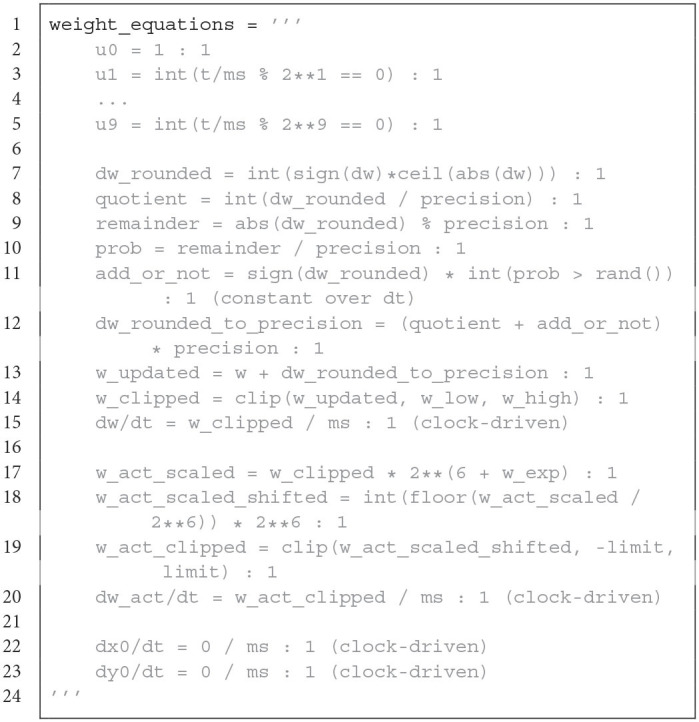
Weight equations for Brian. The first part creates variables that allow terms of the plasticity rule to be evaluated only at the 2^*k*^ time step. *dw* contains the user defined learning rule. The updated weight mantissa is adapted depending on the number of weight bits, which determines the precision. The weight mantissa is rounded with *stochastic rounding*. After clipping, the weight mantissa is updated and the actual weight is calculated.

#### 4.1.4. State monitor and Spike monitor

The LoihiStateMonitor class extends the StateMonitor class from Brian, while the LoihiSpikeMonitor class extends the SpikeMonitor class. Both classes support the most important parameters from their subclasses and update the schedule for the timing of the probes. This schedule update avoids shifts in the monitored variables compared to Loihi.

#### 4.1.5. Spike generator group

The LoihiSpikeGeneratorGroup extends the SpikeGeneratorGroup class from Brian. This class only reduces the available parameters to avoid that users unintentionally change variables which would cause an unwanted emulation behavior.

### 4.2. Examples

To demonstrate that the Loihi emulator works as expected, we provide three examples covering a single neuron, a recurrently connected spiking neural network, and the application of a learning rule. All three examples are available as Jupyter notebooks.^8^

#### 4.2.1. Neuron model

In a first test, we simulated a single neuron. The neuron receives randomly timed excitatory and inhibitory input spikes. Figure 1A1 shows the synaptic responses induced by the input spikes for the simulation using the Loihi chip and the Loihi emulator. The corresponding voltage traces are shown in Figure 1A2. As expected, the synaptic input as well as the voltage match perfectly between the hardware and the emulator.

#### 4.2.2. Network

In a second approach we applied a recurrently connected network of 400 excitatory and 100 inhibitory neurons with log-normal weights. The network gets noisy background input from 40 Poisson generators that are connected to the network with a probability of 0.05. As already shown by others, this setup leads to a highly chaotic behavior (Sompolinsky et al., 1988; Van Vreeswijk and Sompolinsky, 1996; Brunel, 2000; London et al., 2010). Despite the chaotic dynamics, spikes, voltages and synaptic inputs match perfectly for all neurons and over the whole time. The spiking pattern of the network is shown in Figure 2B. All yellow (Brian) and blue (Loihi) dots match perfectly.

#### 4.2.3. Learning

In the last experiment, we applied a simple STDP learning rule, as introduced in Equation (25), at a single plastic synapse. The experiment is sketched in Figure 2A. One spike generator, denoted *input*, has a plastic connection to a neuron with a very low weight ($\tilde{w}=128$, Θ = −6), such that it has a negligible effect on the post-synaptic neuron. Another spike generator, denoted *noise*, has a large but static weight ($\tilde{w}=254$, Θ = 0) to reliably induce post-synaptic spikes. Figure 2B compares the distribution of traces between the emulator and Loihi. For this 400 trials were simulated.

We chose an asymmetric learning window for the STDP rule. The learning rule uses one pre-synaptic trace *x*_1_ (${\hat{x}}_{1}=120$, τ_*x*_1__ = 8) and one post-synaptic trace *y*_1_ (ŷ_1_ = 120, τ_*y*_1__ = 8). In addition the dependency factors *x*_0_ ∈ 0, 1 and *y*_0_ ∈ 0, 1 are used, which indicate a pre- and post-synaptic spike respectively. Using these components, the learning rule is defined as


(25) $$\begin{array}{l} dw=2^{-2}\cdot x_{1}\cdot y_{0}-2^{-2}\cdot x_{0}\cdot y_{1}. \end{array}$$

Due to the stochastic rounding of the traces, differences in the weight changes occur, which are shown in Figure 2C. Fortunately, the relative weight error remains low at a constant level of 0.027 ± 0.027 and does not diverge, even over long simulation times, e.g., 100000 steps. Despite these variations, the STDP learning window of the emulator reproduces the behavior of the Loihi learning window, as shown in Figure 2D.

### 4.3. Performance tests

An important argument for the development of the Brian2Loihi emulator was—besides improving the understanding of Loihi's functionality—its usefulness for prototyping. When developing new models, algorithms, and applications, often large parameter scans are performed in which many networks with different parameter sets are initialized and executed. During this process, it is crucial to be able to read out spiking information to measure performance. For this reason we measured initialization and execution times both with and without spike monitoring on the Loihi chip and in the Loihi emulator.

**Figure 3:**
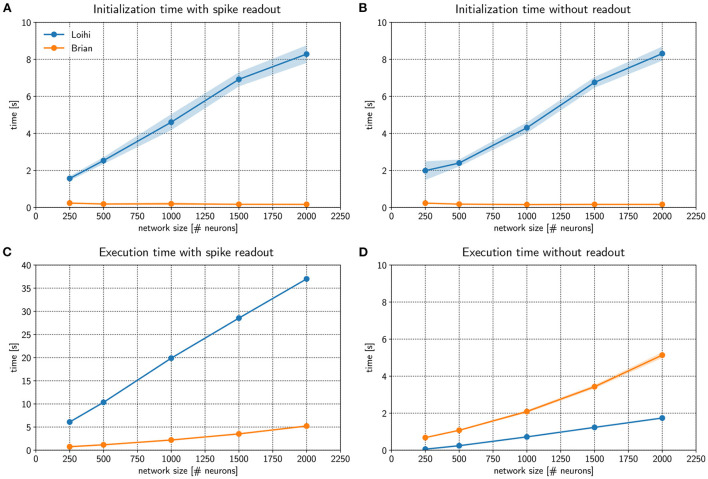
Comparing performance of the Loihi emulator with the Loihi chip. The initialization time **(A,B)** and execution time **(C,D)** for Loihi-based (blue) and emulator-based (orange) simulations was compared for different network sizes. In one case spikes were read out for all neurons and time steps **(A,C)** and in a second case no spikes were read out **(B,D)**. The Brian-based simulation using Brian2Loihi had faster initialization times across all network sizes, both with and without spike monitoring. For the execution time, a Brian-based simulation was only faster when a read out was defined. If no spikes were read out, Loihi-based execution is faster. Execution time both on the Loihi chip and using the emulator increase with network size. Points show the mean and shaded areas show the standard deviation over 5 trials.

Figure 3A compares *initialization* times for a randomly connected network with different sizes. Networks were stimulated with background noise to maintain a consistent firing rate. Note that more details about the network implementation are provided in Appendix 9.2.2. From the figure, it is clear that Loihi takes much more time to setup the network compared to the emulator based on Brian. If no spiking information is read out from the network during simulation, the result is quite similar, as shown in Figure 3B. Brian2Loihi reduces the initialization time drastically, in particular for larger networks. This boost in initialization time is highly valuable for parameter scans across many network configurations.

We were also interested in the comparison for the *execution* times of the simulation. Figure 3C compares Loihi- and Brian-based execution times if all spikes were read out. Clearly, Brian2Loihi is much faster and the difference becomes larger as the network size grows. However, if no read out is performed, Figure 3D shows that in this case Loihi is faster in executing the simulation across all network sizes. Therefore, Brian2Loihi is more efficient for prototyping networks, when we depend on analyzing comprehensive data from the networks' behavior. For applications where a read out is not important or only few spikes must be read out, execution on Loihi is faster.

This underlines the significance of the Brian2Loihi emulator for prototyping on one hand and shows the potential of Loihi for large and long-term network simulations on the other hand. Note, however, that due to longer initialization times on Loihi, faster execution times are likely beneficial only if network initialization must not be performed often, readout is minimal, and the simulation time is long. In many cases, choosing a Brian-based simulation for development and a Loihi-based simulation for productive use cases could be an efficient combination in our view.

### 4.4. Applications

As a starting point for working with the emulator beyond the examples above, here we briefly describe two more complex applications implemented using the emulator. The code is openly available.

#### 4.4.1. Anisotropic network

In a recent study, we showed that a recurrently connected neural network with spatially inhomogeneous locally correlated connectivity (i.e., “the anisotropic network”, for original model see Spreizer et al., 2019) could be implemented on Loihi to generate noise-robust trajectories for robotic movements (Michaelis et al., 2020). This biologically plausible network model can generate stable sequences of neural activity on the timescale of behavior, making it interesting for both neuroscience and for neuromorphic applications. We implemented this network in the Loihi emulator and made it publicly available on GitHub.^9^

#### 4.4.2. SSSP

The goal of the Single Source Shortest Path (SSSP) problem is to find the shortest path from a start node to a target node in a given graph. Spiking neuronal networks can solve the problem through a wave front algorithm (Ponulak and Hopfield, 2013). Within this algorithm a wave of spikes propagates through a network of neurons that acts to represent the graph. The algorithm stops when the target neuron spikes. To enable path back tracing a local learning rule alters the weights during the wave propagation phase accordingly. An implementation using the Loihi emulator is available on GitHub.^10^

Furthermore, a new type of the SSSP algorithm for neuromorphic hardware was developed using the Loihi emulator, the so-called add-and-minimize (AM) algorithm (Michaelis, 2022, Appendix 9.5). It is capable of solving the SSSP problem for larger graphs, especially when the costs of the edges have a higher resolution. The code is again publicly available.^11^

## 5. Discussion

This study was motivated by two goals. We hope to simplify the transfer of models to Loihi and therefore developed a Loihi emulator for Brian, featuring many functionalities of the Loihi chip. In the process of developing the emulator, we aimed to provide a deeper understanding of the functionality of the neuromorphic research chip Loihi by analyzing its neuron and synapse model, as well as synaptic plasticity.

We hope that the analysis of Loihi's spiking units has provided some insight into how Loihi computes. With the numerical integration method, numerical precision and related rounding method, as well as the update schedule, we were able to walk from the LIF neuron model down to the computations performed. For neurons and networks without plasticity we are able to emulate Loihi without error. Analyzing and implementing synaptic plasticity showed that, due to stochastic rounding, it is not possible to exactly replicate trial by trial behavior when it comes to learning. However, on average the weight changes induced by a learning rule are preserved.

The main benefit of the Brian2Loihi emulator lies in lowering the hurdle for the experimenter. Especially in neuroscience, many scientists are accustomed to neuron simulators and in particular Brian is widely used. It makes a deep dive into new software frameworks and hardware systems unnecessary. The emulator can be used for simple and fast prototyping, as it improves the initialization time in all cases drastically and the execution time, when a read out is used. In addition, hardware specific complications, like distributing neurons to cores, or constraints like potential limits on the number of available neurons or synapses, or on the speed or size of read-out, do not occur in the emulator. While this will surely improve with new generations of hardware and software in the upcoming years, they can already be ignored by using the emulator.

At this point it is important to note that not all Loihi features are included in the emulator, yet. In particular, the homeostasis mechanism, rewards, and tags for the learning rule are not included. In Table A1, we provide a comparison of all functionalities from Loihi with those available in the current state of the emulator. Development of this emulator is an open source project and we expect improvements and additions with time. Note that a follow up project, called Brian2Lava has already started.^12^

An important vision for the future is to flexibly connect front-end development environments (e.g., Brian, NEST, Keras, TensorFlow) with various back-ends, like neuromorphic platforms (e.g., Loihi, SpiNNaker, BrainScaleS, Dynap-SE) or emulators for these platforms. PyNN (Davison et al., 2009) is such an approach to unify different front-ends and back-ends in a more general way. Nengo (Bekolay et al., 2014), as another approach, does not provide the use of other simulators, but allows several back-ends and focuses on higher level applications (DeWolf et al., 2020). NxTF (Rueckauer et al., 2021) is an API and compiler aimed at simplifying the efficient deployment of deep convolutional spiking neural networks on Loihi using an interface derived from Keras. We think that ideally, one could continue to work in their preferred front-end environment while a package maps their code to existing chips or computer-based emulators of these chips. We expect an interface along these lines will play an important role in the future of neuromorphic computing and want to contribute to this development with our Brian2Loihi emulator.

At least for now, with an emulator at hand, it is easier to prototype network models and assess whether an implementation on Loihi is worth considering. When getting started with neuromorphic hardware, to e.g., scale up models or speed up simulations, researchers familiar with Brian can directly deploy models prepared with the emulator. We hope that with this, others may find a smooth entry into the quickly emerging field of neuromorphic computing.

## Data availability statement

The original contributions presented in the study are included in the article/Supplementary material, further inquiries can be directed to the corresponding author/s.

## Author contributions

CM, AL, and WO analyzed Loihi's neuron and synapse model, with a larger contribution from AL and tested and refined the emulator implementation. CM programmed the emulator. WO performed the simulations and created the main figures and edited and reviewed. CM and AL created the supplementary figures and wrote the text. CT acquired funding and supervised the study. All authors reviewed the manuscript. All authors contributed to the article and approved the submitted version.

## Funding

This study received funding from Intel Corporation. The funder was not involved in the study design, collection, analysis, interpretation of data, the writing of this article or the decision to submit it for publication.

## Conflict of interest

The authors declare that the research was conducted in the absence of any commercial or financial relationships that could be construed as a potential conflict of interest.

## Publisher's note

All claims expressed in this article are solely those of the authors and do not necessarily represent those of their affiliated organizations, or those of the publisher, the editors and the reviewers. Any product that may be evaluated in this article, or claim that may be made by its manufacturer, is not guaranteed or endorsed by the publisher.

## Footnotes

^1^ https://www.nengo.ai/nengo-loihi/

^2^ https://code.ini.uzh.ch/yigit/NICE-workshop-2021

^3^ https://github.com/andrewlehr/loihi_parameter_tuning_dashboard

^4^The documentation for the NxSDK is available from Intel on request.

^5^ https://github.com/sagacitysite/brian2_loihi_utils/blob/main/algorithm/02_weight-calculation.ipynb

^6^ https://pypi.org/project/brian2-loihi/

^7^ https://github.com/sagacitysite/brian2_loihi/

^8^ https://github.com/sagacitysite/brian2_loihi_utils/tree/main/examples

^9^ https://github.com/andrewlehr/Brian2Loihi_SpreizerNet

^10^ https://github.com/Winnus/Brian2Loihi_SSSP

^11^ https://github.com/elena-off/sssp-loihiemulator

^12^ https://gitlab.com/tetzlab/brian2lava
