# Supplementary material for: Brian2Loihi: An emulator for the neuromorphic chip Loihi using the spiking neural network simulator Brian
Source: Front Neuroinform. 2022 Nov 9;16:1015624. doi: 10.3389/fninf.2022.1015624 (PMC9682266; doi:10.3389/fninf.2022.1015624)
Supplement: Supplementary file 1 [file Data_Sheet_1.pdf]

Appendix

of

Brian2Loihi: An emulator for the neuromorphic chip Loihi using the spiking neural network simulator Brian

Carlo Michaelis[1,2,*], Andrew B. Lehr[1,2,*], Winfried Oed[1,2,*], and Christian Tetzlaff[1,2]

[1]Department of Computational Neuroscience, University of Göttingen, Germany
[2]Bernstein Center for Computational Neuroscience, University of Göttingen, Germany
[*]These authors contributed equally.

9 Appendix

9.1 Loihi neuron model

Computational units on `Loihi` communicate via spikes. They can be connected up to form networks, each unit both sending and receiving spikes from some subset of the other units. Like neurons in the brain, a unit emits a spike if its internal variable reaches a certain threshold. The spike is then transmitted to all units with a direct incoming connection from the one that spiked. This induces a change in the receiving units' internal variable. At every time step, the internal variable of all units' decays towards zero, counteracting any input received. And after spiking, the internal variable is reset to zero. In terms of the brain, each computational unit on `Loihi` implements a simple model of a spiking neuron, in particular a variant of the leaky integrate and fire neuron model, which is based on a simple resistor-capacitor (RC) circuit. Readers are encouraged to consult the first chapter of Gerstner et al. (2014) for a more detailed treatment.

9.1.1 Voltage

We refer to the standard leaky integrate and fire neuron, as it is defined in Gerstner et al. (2014). In this model, the difference in electric potential between the interior and the exterior of a neuron, the so-called membrane potential, evolves according to

$$\tau_v \frac{dv}{dt} = -[v(t) - v_{rest}] + RI(t), \tag{1}$$

where v is the voltage across the membrane, τ_v is the membrane time constant of the neuron, v_{rest} is the resting potential, I is the input current and R is the resistance of the membrane. Whenever the membrane potential reaches threshold v_i^{th} it is reset to v_{rest}.

Davies et al. (2018) present the following variant of the standard LIF model which forms the basis for `Loihi`'s computational units

$$\frac{dv_i}{dt} = -\frac{1}{\tau_v} v_i(t) + I_i(t) - v_i^{th} \sigma_i(t), \tag{2}$$

where v is the voltage across the membrane, τ_v is the time constant for voltage decay, I is in this case an input variable, v_i^{th} is the threshold voltage to spike, and $\sigma(t)$ indicates whether the neuron fired a spike at time t.

There are a few differences that are worth noting. In the `Loihi` variant, the resting potential is zero. The membrane time constant τ_v applies only to the voltage decay and not to the input variable I. In effect, the resistance and the time constant are implicit in the input variable I as connection weight.

Further, resetting after a spike is included directly in the differential equation. The final term subtracts the threshold voltage v_i^{th} at the time of a spike. This is a matter of notation and can be written as such, or with a separate reset condition $v_i \to 0$ applied at the time of each spike, as in Gerstner et al. (2014).

9.1.2 Derivation of synaptic response

To keep this paper self-contained, here we derive the synaptic response of a `Loihi` unit to an incoming spike train. For the reader's convenience, we repeat the definition from Equation **??** in the main text here and then with a few steps obtain the result from Equation **??**.

Definition. The *synaptic response* is given by

$$I_i(t) = \sum_j J_{ij} (\alpha_I * \sigma_j)(t) + I_i^{\text{bias}}, \tag{3}$$

where J_{ij} is the weight from unit j to i, I_i^{bias} is a constant bias input, and the spike train σ_j of unit j is convolved with the *synaptic filter impulse response* α_I, given by

$$\alpha_I(t) = \exp\left(-\frac{t}{\tau_I}\right) H(t), \tag{4}$$

where τ_I is the time constant of the synaptic response and $H(t)$ the unit step function. Note we define $\alpha_I(t)$ differently here than in Davies et al. (2018) (see Appendix 9.1.3 for details).

Definition. The *unit step function* $H : \mathbb{R} \to \mathbb{R}$ is given by

$$H(x) = \begin{cases} 1, & x \geq 0 \\ 0, & x < 0. \end{cases} \tag{5}$$

Definition. The *Dirac delta* is a tempered distribution $\delta \in \mathcal{S}'(\mathbb{R})$, with $\delta : \mathcal{S}(\mathbb{R}) \to \mathbb{C},\ \varphi \mapsto \langle \delta, \varphi \rangle$ where

$$\langle \delta, \varphi \rangle := \int_{-\infty}^{\infty} \delta(x) \varphi(x)\, dx := \varphi(0) \tag{6}$$

for all Schwartz functions $\varphi \in \mathcal{S}(\mathbb{R})$. Here we extend the definition such that $\delta : f \to f(0)$ for arbitrary, everywhere-defined $f : \mathbb{R} \to \mathbb{R}$.

Definition. We define the translation of δ by a, denoted δ_a, as the distribution $\tau_a \delta : \mathcal{S}(\mathbb{R}) \to \mathbb{C}$ with

$$\tau_a \delta(\varphi) := \langle \delta_a, \varphi \rangle = \int_{-\infty}^{\infty} \delta(x - a) \varphi(x)\, dx \tag{7}$$

and again extend this notion to arbitrary, everywhere-defined $f : \mathbb{R} \to \mathbb{R}$.

Lemma 1. (*translation property*) $\tau_a \delta(f) = f(a)$, for $a \in \mathbb{R}$ and $f : \mathbb{R} \to \mathbb{R}$.

Proof. Let $f : \mathbb{R} \to \mathbb{R}$. Then

$$\tau_a \delta(f) = \int_{-\infty}^{\infty} \delta(x-a) f(x)\, dx = \int_{-\infty}^{\infty} \delta(x) f(x+a)\, dx = f(0+a) = f(a) \tag{8}$$

■

Corollary 1. As a sum of Dirac deltas, σ_i can be understood as the following linear functional

$$\sigma_i := \sum_k \tau_{t_{i,k}} \delta \; : \; \varphi \mapsto \mathbb{C}, \tag{9}$$

with

$$\langle \sigma_i, \varphi \rangle := \langle \sum_k \delta_{t_{i,k}}, \varphi \rangle = \sum_k \langle \delta_{t_{i,k}}, \varphi \rangle = \sum_k \varphi(t_{i,k}), \qquad \varphi \in \mathcal{S}(\mathbb{R}), \tag{10}$$

and again we extend this notion from the space of tempered distributions to σ_i for arbitrary, everywhere-defined f.

Definition. The *convolution* between the Dirac delta distribution and a function is to be understood in the following sense

$$(\delta * f)(x) := \langle \delta, \tau_x \tilde{f} \rangle = \int_{-\infty}^{\infty} \delta(y) f(x-y)\, dy = \int_{-\infty}^{\infty} \delta(x-y) f(y)\, dy \tag{11}$$

where $\tilde{f}(x) = f(-x)$.

Lemma 2. $(\delta * f)(x) = f(x)$.

Proof. Using $\delta(x) = \delta(-x)$ (E) and the translation property of the Dirac delta function (T) from Lemma 1 we have

$$(\delta * f)(x) := \int_{-\infty}^{\infty} \delta(x-y) f(y)\, dy \overset{E}{=} \int_{-\infty}^{\infty} \delta(y-x) f(y)\, dy = \tau_x \delta(f) \overset{T}{=} f(x). \tag{12}$$

■

Claim. The synaptic input $I_i(t)$ for unit i is given by

$$I_i(t) = \sum_j J_{ij} \sum_k \exp\left(\frac{t_{j,k} - t}{\tau_I}\right) H(t - t_{j,k}) + I_i^{\text{bias}}.$$

Proof. Applying the definition of convolution (D), linearity of the integral operator (L), the translation property of the Dirac delta function (T), and using that $\delta(x) = \delta(-x)$ (E) we have

$$(\alpha_I * \sigma_j)(t) \overset{D}{=} \int_{-\infty}^{\infty} \alpha_I(s)\, \sigma_j(t-s)\, ds \tag{13}$$

$$= \int_{-\infty}^{\infty} \alpha_I(s) \sum_k \delta(t - t_{j,k} - s)\, ds \tag{14}$$

$$\overset{L}{=} \sum_k \int_{-\infty}^{\infty} \alpha_I(s) \delta(t - t_{j,k} - s)\, ds \tag{15}$$

$$\overset{E}{=} \sum_k \int_{-\infty}^{\infty} \alpha_I(s) \delta(s - (t - t_{j,k}))\, ds \tag{16}$$

$$= \sum_k \tau_{t - t_{j,k}} \delta(\alpha_I) \tag{17}$$

$$\overset{T}{=} \sum_k \alpha_I(t - t_{j,k}) \tag{18}$$

With this, we can write the synaptic input (Equation ??) as

$$I_i(t) = \sum_j J_{ij} \sum_k \alpha_I(t - t_{i,k}) + I_i^{\text{bias}} \tag{19}$$

$$= \sum_j J_{ij} \sum_k \exp\left(\frac{t_{j,k} - t}{\tau_I}\right) H(t - t_{j,k}) + I_i^{\text{bias}}. \tag{20}$$

■

We see the input can be written as a sum of exponentially decaying functions with amplitude J_{ij} beginning at the time of each spike $t_{j,k}$.

9.1.3 Definition of the synaptic filter impulse response

Davies et al. (2018) defined the *synaptic filter impulse response* as

$$\alpha_I^{orig}(t) = \frac{1}{\tau_I} \exp\left(-\frac{t}{\tau_I}\right) H(t). \tag{21}$$

Note that we have omitted the factor of $1/\tau_I$ in our definition, in particular we defined

$$\alpha_I(t) = \exp\left(-\frac{t}{\tau_I}\right) H(t). \tag{22}$$

We prefer this formulation as the results obtained match exactly with the `Loihi` documentation. If, however, the factor of $1/\tau_I$ is included, the factor is carried through to Equation ??. Namely it becomes

$$I[t] = I[t-1] \cdot (2^{12} - \delta^I) \cdot 2^{-12} + \frac{J}{\tau_I} \cdot s[t] \tag{23}$$

where we see there is an extra factor of $1/\tau_I$ multiplied by the weight J. The definition from Davies et al. (2018) and the `NxSDK` documentation can be reconciled by replacing this extra factor of $1/\tau_I$ with a static factor 2^6 and then considering the weight to be $J = \tilde{w} \cdot 2^{\Theta}$ instead of $J = \tilde{w} \cdot 2^{6+\Theta}$.

9.2 Miscellaneous implementational details

9.2.1 Brian state update schedule

In `Brian` the `network` class is the main class of a simulation. All containing objects like neurons, synapses, monitors, poisson generators, are added to that `network` object. Each of these objects have a *when* attribute. The `network` class decides in which order containing objects are updated depending on their *when* attribute. For this decision a schedule is defined, given as a string list. The default schedule is `['start', 'groups', 'thresholds', 'synapses', 'resets', 'end']`.

We observed that `Loihi` implements a schedule where first the synapses are updated and afterwards the neuron groups. In `Brian` the evaluation is performed in opposite order, which results in a shift between `Loihi` and the emulator. We therefore changed the `Brian` schedule to `['start', 'synapses', 'groups', 'thresholds', 'resets', 'end']`, i.e. the synapse update is pulled in front of groups.

Additionally the time when the synaptic monitor is evaluated is different in `Loihi`. For the emulator, we also needed to adjust these. This is done by changing the monitors *when* flag from the default *start* to *synapses* for the synaptic input and all pre- and post-synaptic trace variables. For probing the voltage

and weight the *when* attribute was changed to *end*. The same holds for probing spikes with the spike monitor. Moreover, the poisson generators *when* flag has to be changed from the default *thresholds* to *start* to ensure Poisson spikes are given at the beginning of the current time step and are propagated through the simulation schedule.

9.2.2 Voltage memory used to count refractory time

Note that `Loihi` sets the voltage of a neuron to a non zero value if the neuron has spiked. The memory for storing the voltage is used for counting while the neuron is in refractory state. This causes a deviation between the emulator and `Loihi` for the voltage, which is only due to technical reasons and has no functional effect.

9.3 Further details on performance tests

In order to compare the speed between `Loihi`- and `Brian`-based simulations, randomly connected networks with different sizes were simulated for 1000 time steps. Here, we give details about the implementation of these networks, results are described in Section **??**.

All networks consist of 80% excitatory and 20% inhibitory neurons. Every neuron was connected to 40 other neurons in average. The synaptic weights were randomly drawn from a log-normal distribution. The weights were generated using the `PeleNet` framework (Michaelis, 2020) and copied to the emulator to ensure that the weights are equal for both systems. The excitatory neurons within the networks were stimulated with sufficient background noise to drive spiking such that the mean firing rate was consistent across networks. For this, noise neurons were defined that generate Poisson spikes. The amount of noise neurons was 10% of the excitatory neurons in the network. To keep a similar spiking behavior for all networks, independent of their size, the connectivity between the noise neurons and the excitatory neurons was chosen as $^{40}/_{n}$, where n is the number of excitatory neurons. With this, the networks had a relatively constant overall mean firing rate of $0.251\,^{1}/_{s} \pm 0.017\,^{1}/_{s}$.

The simulation times were separated into initialization time and execution time. For this we obtained the execution time for the `Brian`-based and `Loihi`-based simulations with their respective analysis tools. The `NxSDK` directly provides initialization times. For `Brian`, we took the time needed to run the whole `run()` command and subtracted the execution time. The difference was considered as initialization time. Note that the time for executing the network definitions was not considered for both `Brian` and the `NxSDK`.

9.4 Plastic weight update with stochastic rounding

If the weight is updated by a learning rule, the weight mantissa needs to be updated according to the given precision, as described in Section **??**. The precision is determined by the available number of bits, which can be chosen by the user, and in addition depends on the sign mode. To test the implementation in the emulator, we compared its behavior to `Loihi` for each possible number of weight bits. In particular, for an excitatory plastic synapse we increased the weight mantissa by one at each time step (via learning rule $dw = u_0$) and measured the actual weight after the update (i.e. rounding and shifting). Our expectation was that for *stochastic rounding* to the nearest 2^{n_s}, the average number of time steps required until a weight change takes place should be equal to 2^{n_s}. This is because the probability of rounding up from a given weight mantissa, e.g. $\tilde{w} := k \cdot 2^{n_s}$, when 1 is added can be calculated from Equation **??** as

$$(|w| - \lfloor |w| \rfloor_{2^{n_s}}))/2^{n_s} = ((k \cdot 2^{n_s} + 1) + k \cdot 2^{n_s})/k \cdot 2^{n_s} = 1/2^{n_s}.$$

As expected, the results match `Loihi`'s behavior nicely, as seen in Figure 1, confirming the validity of our implementation.

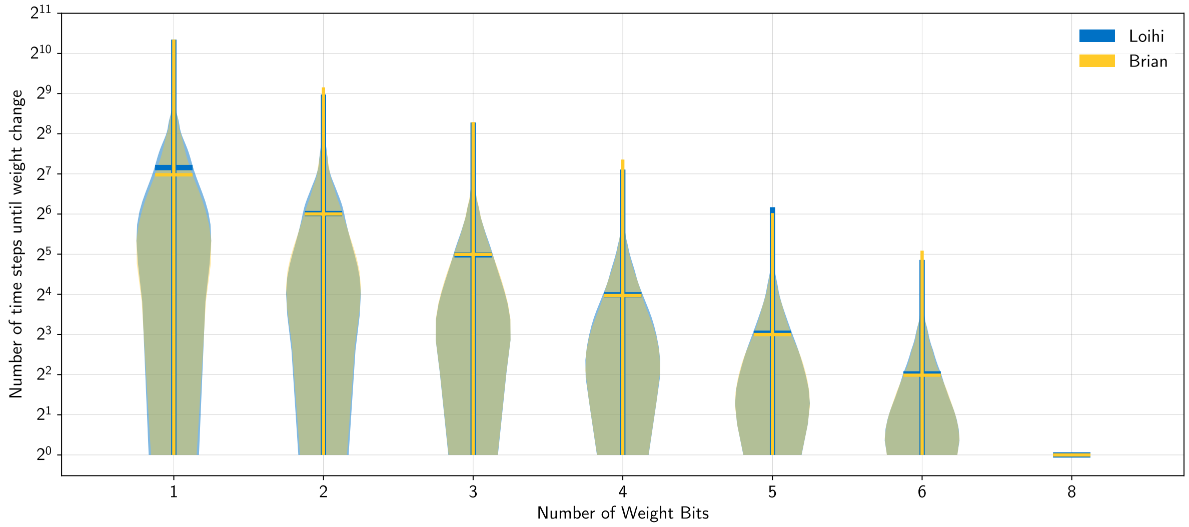

Figure 1: Distribution of the weight change for different number of weight bits. The weight mantissa is increased by 1 in every time step. Due to stochastic rounding, this change may then be rounded up or down. Shown is the distribution of the number of time steps until a weight change occurs. For each number of weight bits, 8000 weight changes were sampled for `Loihi` and the emulator. The emulator implementation matches `Loihi` well.

9.5 Pre- and post-synaptic decay deviations

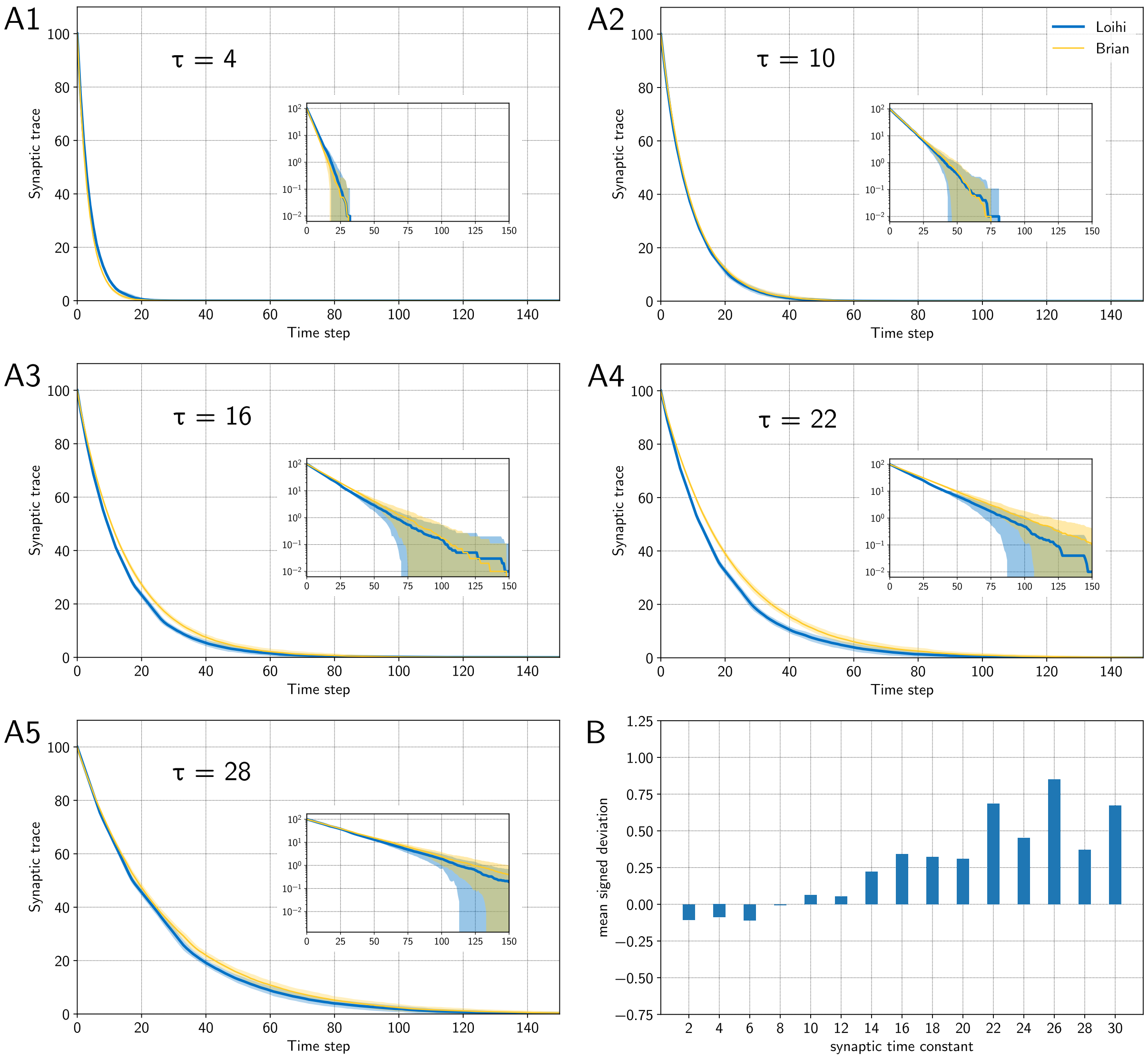

Figure 2: Deviations of the synaptic traces between `Loihi` and the emulator. **A** Synaptic traces for different synaptic time constants τ. Averaged over 100 trials each. The inlay shows the traces in a logarithmic scale. Blue indicates the trace from `Loihi`, yellow the trace from the emulator. **B** Mean signed deviation for different synaptic time constants *tau* over 100 trials each. For low *tau* values, the emulator is slightly below the `Loihi` reference, whereas it lies slightly above the `Loihi` traces for higher values.

9.6 Emulator features

**Loihi**	**Emulator**
neurons	
current impulse/decay	✓
voltage impulse/decay	✓
bias input	(✓)
homeostasis (threshold adaption)	-
random noise for current	-
random noise for voltage	(✓)
multi-compartment neurons	(✓)
connections	
weight mantissa/exponent	✓
weight precision	✓
synaptic delay	✓
box-synapse	-
learning	
presynaptic spike	✓
1st presynaptic trace	✓
2nd presynaptic trace	✓
postsynaptic spike	✓
1st postsynaptic trace	✓
2nd postsynaptic trace	✓
3nd postsynaptic trace	✓
synaptic weight as variable	✓
reward spike	-
reward trace	-
tag	-
plastic synaptic delay	-
learning epoch	(✓)
probes	
probe variables	✓
probing conditions	(✓)

Table 1: Features of `Loihi` compared with the emulator (version 0.5.2). Check marks in brackets are not fully supported or can manually be included using core `Brian` functionality.

References

Davies, M., Srinivasa, N., Lin, T., Chinya, G., Cao, Y., Choday, S. H., Dimou, G., Joshi, P., Imam, N., Jain, S., Liao, Y., Lin, C., Lines, A., Liu, R., Mathaikutty, D., McCoy, S., Paul, A., Tse, J., Venkataramanan, G., . . . Wang, H. (2018). Loihi: A neuromorphic manycore processor with on-chip learning. *IEEE Micro*, *38*(1), 82–99. https://doi.org/10.1109/MM.2018.112130359

Gerstner, W., Kistler, W. M., Naud, R., & Paninski, L. (2014). *Neuronal dynamics: From single neurons to networks and models of cognition*. Cambridge University Press.

Michaelis, C. (2020). Pelenet: A reservoir computing framework for loihi. https://doi.org/10.48550/ARXIV.2011.12338
